# Bioinformatics Describes Novel Loci for High Resolution Discrimination of *Leptospira* Isolates

**DOI:** 10.1371/journal.pone.0015335

**Published:** 2010-10-15

**Authors:** Gustavo M. Cerqueira, Alan J. A. McBride, Rudy A. Hartskeerl, Niyaz Ahmed, Odir A. Dellagostin, Marcus R. Eslabão, Ana L. T. O. Nascimento

**Affiliations:** 1 Centro de Biotecnologia, Instituto Butantan, São Paulo, Brazil; 2 Gonçalo Moniz Institute, Oswaldo Cruz Foundation, Salvador, Brazil; 3 Department of Biomedical Research, Royal Tropical Institute, World Health Organization/Food and Agriculture Organization of the United Nations/World Organisation for Animal Health (WHO/FAO/OIE) and National Leptospirosis Reference Centre, Amsterdam, The Netherlands; 4 Pathogen Biology Laboratory, Department of Biotechnology, University of Hyderabad, Hyderabad, India; 5 Centro de Biotecnologia, Universidade Federal de Pelotas, Pelotas, Brazil; 6 Interunidades em Biotecnologia, Instituto de Ciências Biomédicas, USP, São Paulo, Brazil; Charité-Universitätsmedizin Berlin, Germany

## Abstract

**Background:**

Leptospirosis is one of the most widespread zoonoses in the world and with over 260 pathogenic serovars there is an urgent need for a molecular system of classification. The development of multilocus sequence typing (MLST) schemes for *Leptospira* spp. is addressing this issue. The aim of this study was to identify loci with potential to enhance *Leptospira* strain discrimination by sequencing-based methods.

**Methodology and Principal Findings:**

We used bioinformatics to evaluate pre-existing loci with the potential to increase the discrimination of outbreak strains. Previously deposited sequence data were evaluated by phylogenetic analyses using either single or concatenated sequences. We identified and evaluated the applicability of the *ligB*, *secY*, *rpoB* and *lipL41* loci, individually and in combination, to discriminate between 38 pathogenic *Leptospira* strains and to cluster them according to the species they belonged to. Pairwise identity among the loci ranged from 82.0–92.0%, while interspecies identity was 97.7–98.5%. Using the *ligB-secY-rpoB-lipL41* superlocus it was possible to discriminate 34/38 strains, which belong to six pathogenic *Leptospira* species. In addition, the sequences were concatenated with the superloci from 16 sequence types from a previous MLST scheme employed to study the association of a leptospiral clone with an outbreak of human leptospirosis in Thailand. Their use enhanced the discriminative power of the existing scheme. The *lipL41* and *rpoB* loci raised the resolution from 81.0–100%, but the enhanced scheme still remains limited to the *L. interrogans* and *L. kirschneri* species.

**Conclusions:**

As the first aim of our study, the *ligB-secY-rpoB-lipL41* superlocus demonstrated a satisfactory level of discrimination among the strains evaluated. Second, the inclusion of the *rpoB* and *lipL41* loci to a MLST scheme provided high resolution for discrimination of strains within *L. interrogans* and *L. kirschneri* and might be useful in future epidemiological studies.

## Introduction

Leptospirosis is a zoonotic disease caused by pathogenic *Leptospira* spp. and is considered an emerging global public health problem [1], [2]. Furthermore, the impact of leptospirosis has increased, particularly in poverty stricken regions of the world, due to the high mortality (>50%) associated with the recent increase of severe pulmonary haemorrhage syndrome (SPHS) in patients with severe leptospirosis [3], [4]. Based on serology, *Leptospira* spp. are traditionally classified into 29 serogroups and over 300 serovars [5]–[7]. More recently, genetic methods have attempted to replace the traditional classification methods and DNA-DNA hybridization studies have identified 20 *Leptospira* spp. to date [5], [8]–[11]. Several typing methods have been employed to classify isolates with differing degrees of success [12]. However, a major limitation is the lack of correlation between the serologic and genotypic classification methods [5], [12], [13].

Multilocus sequence typing (MLST) was originally developed for bacteria using *Neisseria meningitidis* isolates [14] and, so far, it has been successfully applied to over 30 bacteria [15], [16]. In the field of leptospirosis, efforts to develop a typing method have focused on MLST [17], [18]. MLST allows the adoption of a universal format for a particular bacterial species and permits the sequence data generated to be easily exchanged over the Internet. Traditionally, the loci chosen for MLST analyses are based on 6–10 housekeeping genes that are under selection for metabolic functionality [15]. Since this group is comprised of slowly evolving genes they are likely to be more conserved and stable within a particular species [19]. Unfortunately it has not been possible to identify a set of housekeeping genes with universal applicability to all bacterial pathogens. Rather, MLST loci are chosen empirically and evaluated for each individual pathogen [15]. Ahmed and colleagues presented the first MLST scheme based on loci from four housekeeping genes and two genes encoding outer-membrane proteins for typing *L. alexanderi*, *L. borgpetersenii*, *L. interrogans*, *L. kirschneri*, *L. noguchii* and *L. santarosai* isolates [18]. An alternative MLST scheme using loci from seven housekeeping genes was used to type *L. interrogans* and *L. kirschneri* isolates and is available on the Internet (http://leptospira.mlst.net/). The database contains 109 sequence types (ST) and sequences from 263 isolates at time of writing [17]. Although this evidently moved the field forward, a limitation of this database is that it only applies to isolates from two *Leptospira* species, *L. interrogans* and *L. kirschneri*. The ideal MLST scheme should be valid for all *Leptospira* spp. or at least the pathogenic species [20], and provide discrimination beyond the species level [21].

High-resolution typing, such as that required during outbreak investigations, usually requires the inclusion of genes with greater diversity, e.g. antigen genes, rather than housekeeping genes [15]. The objective of this study was to carry out a bioinformatics-based analysis of *Leptospira* genes available in GenBank to identify potential targets for improved *Leptospira* discrimination. The genes *ligB*, *secY*, *lipL41* and *rpoB* were identified as potential genes for use in an improved typing scheme.

## Methods

### DNA sequences

The DNA sequences for the *ligB*, *secY*, *rpoB* and *lipL41* loci used in this study were obtained from GenBank and LepBank [22] (Table 1) or from the authors of the original *Leptospira* MLST scheme [18]. Most of these sequences were generated by the authors during previous studies and they belong to different reference strains and clinical isolates. The sizes of the loci analyzed were 214 bp (*ligB*), 245 bp (*secY*), 541 bp (*rpoB*) and 884 bp (*lipL41*) and correspond to nucleotide positions 2236–2449 (*ligB*), 771–1015 (*secY*), 1922–2462 (*rpoB*) and 73–956 (*lipL41*). Note that nucleotide positions are based on the *L. interrogans* Copenhageni Fiocruz L1-130 genome (AE016823). These genes can be amplified by using the primers *ligB* (PSBF: 5′-ACWRVHVHRGYWDCCTGGTCYTCTTC-3′; PSBR: 5′-TARRHDGCYBTAATATYCGRWYYTCCTAA-3′), [23]; *secY* (SeqYII: 5′-GAATTTCTCTTTTGATCTTCG-3′; SeqYIV: 5′-GAGTTAGAGCTCAAATCTAAG-3′), [24]; *rpoB* (Lept 1900f: 5′-CCTCATGGGTTCCAACATGCA-3′; Lept 2500r: 5′-CGCATCCTCRAAGTTGTAWCCTT-3′), [25] and *lipL41* (lipL41F: 5′-TAGGAAATTGCGCAGCTACA-3′; lipL41R: 5′-GCATCGAGAGGAATTAACATCA-3′), [18]. The DNA sequences corresponding to the *glmU* (18 alleles), *pntA* (24 alleles), *sucA* (20 alleles), *fadD* (20 alleles), *tpiA* (30 alleles), *pfkB* (35 alleles) and *mreA* (23 alleles) loci were downloaded from the *Leptospira* MLST database at http://leptospira.mlst.net/ [26].

**Table 1:** *Leptospira* serovars and the candidate alleles for MLST.

Species	Serogroup	Serovar	Strain	Accession numbers			
				*ligB*	*secY*	*rpoB*	*lipL41*
*L. borgpetersenii*	Javanica	Ceylonica	Piyasena	EU938500^a^	EU358041^g^	DQ296134^j^	AY461936^m^
	Javanica	Javanica	Veldrat Batavia 46	EU938501^a^	EU358040^g^	DQ296134^j^	AY461938^m^
	Javanica	Poi	Poi	EU938502^a^	EU358007^g^	DQ296134^j^	n/a^ n^
	Mini	Mini	Sari	n/a	EU358032^g^	DQ296134^j^	n/a^ n^
	Sejroe	Hardjo-bovis	JB197	CP000350^b^	CP000350^b^	CP000350^b^	CP000350^b^
	Sejroe	Hardjo-bovis	L550	CP000348^b^	CP000348^b^	CP000348^b^	CP000348^b^
	Tarassovi	Tarassovi	Perepelitsin	n/a	EU358057^g^	EU747307^k^	AY461937^m^
*L. interrogans*	Australis	Australis	Ballico	EU938484^a^	DQ882850^h^	DQ296144^j^	n/a^ n^
	Australis	Bratislava	Jez Bratislava	EU938487^a^	EU357939^g^	EU747300^k^	AY461939^m^
	Australis	Muenchen	Muenchen C90	EU938497^a^	EU357938^g^	DQ296133^j^	n/a^ n^
	Autumnalis	Autumnalis	Akiyami A	EU938485^a^	EU357943^g^	DQ296145^j^	AY461940^m^
	Bataviae	Bataviae	Van Tienen	EU938486^a^	EU357956^g^	DQ296146^j^	AY461941^m^
	Canicola	Canicola	Hond Utrecht IV	EU938488^a^	EU357961^g^	EU747299^k^	AY461942^m^
	Icterohaemorrhagiae	Copenhageni	Fiocruz L1-130	AE016823^c^	AE016823^c^	AE016823^c^	AE016823^c^
	Icterohaemorrhagiae	Icterohaemorrhagiae	RGA	EU938493^a^	EU365950^g^	DQ296133^j^	AY461947^m^
	Icterohaemorrhagiae	Lai	56601	AE010300^d^	AE010300^d^	AE010300^d^	AE010300^d^
	Pyrogenes	Manilae	LT398	EU938496^a^	EU358049^g^	DQ296133^j^	n/a^ n^
	Pyrogenes	Pyrogenes	Salinem	n/a	DQ882863^h^	DQ296147^j^	n/a^ n^
	Sejroe	Hardjo-prajitno	Hardjoprajitno	EU938491^a^	EU357983^g^	EU747303^k^	AY461943^m^
	Sejroe	Wolffi	3705	EU938499^a^	EU357985^g^	EU747308^k^	n/a^ n^
	Hebdomadis	Hebdomadis	Hebdomadis	EU938492^a^	EU357974^g^	EU747304^k^	n/a^ n^
	Pomona	Pomona	Pomona	EU938498^a^	EU358013^g^	EU747306^k^	AY461948^m^
*L. kirschneri*	Australis	Ramisi	Musa	EU938507^a^	EU358020^g^	DQ296139^j^	AY461949^m^
	Autumnalis	Erinaceiauriti	Erinaceus Auritus 670	EU938504^a^	EU358021^g^	DQ296139^j^	AY461950^m^
	Bataviae	Djatzi	HS 26	EU938503^a^	EU358027^g^	DQ296139^j^	AY461951^m^
	Cynopteri	Cynopteri	3522 C	EU938508^a^	EU358027^g^	DQ296139^j^	n/a^ n^
	Grippotyphosa	Grippotyphosa	RM52	AY190126^e^	EU358027^g^	EU747301^k^	AY461953^m^
	Hebdomadis	Kambale	Kambale	EU938505^a^	EU358030^g^	DQ296139^j^	AY461954^m^
	Pomona	Mozdok	5621	EU938506^a^	EU358015^g^	DQ296139^j^	AY461955^m^
*L. noguchii*	Louisiana	Orleans	LSU 2580	EU938509^a^	EU365958^g^	EU349500^l^	AY461957^m^
	Panama	Panama	CZ 214 K	EU938510^a^	EU365958^g^	DQ296141^j^	n/a^ n^
*L. santarosai*	Pyrogenes	Alexi	HS 616	EU938512^a^	EU358047^g^	DQ296131^j^	AY461964^m^
	Sejroe	Trinidad	TRVL 34056	n/a	EU358035^g^	DQ296131^j^	n/a^ n^
	Shermani	Shermani	LT 821	EU938511^a^	DQ882866^h^	DQ296131^j^	AY461965^m^
*L. weilii*	Celledoni	Celledoni	Celledoni	EU938514^a^	EU365960^g^	DQ296132^j^	n/a^ n^
	Hebdomadis	n/a	EcoChallenge	EU700274^f^	AY034036^i^	DQ296132^j^	n/a^ n^
	Javanica	Coxi	Cox	n/a	EU358009^g^	DQ296132^j^	AY461967^m^
	Tarassovi	Vughia	LT 89–68	EU938515^a^	EU365960^g^	DQ296132^j^	AY461968^m^

n/a - Not applicable. Source of sequence data: [26] ^a^; [32] ^b^; [33] ^c^; [34] ^d^; [35] ^e^; [30] ^f^; [28] ^g^; Riediger^h^, unpublished data; [36] ^i^; [29] ^j^; Bomfim^k^, unpublished data; [37] ^l^; [27] ^m^; [18] ^n^.

### Phylogenetic analysis

DNA sequences were aligned using ClustalW at the default settings (http://www.ebi.ac.uk/clustalw). The phylogenetic analyses were performed with Mega 4.1 [27] or Geneious Pro ver 4.7 [28], and the neighbour-joining method with no outgroup. The Tamura-Nei genetic distance model was selected and all trees were resampled using the bootstrap method and 1000 replicates. The phylogenetic trees constructed using sequences of 38 reference strains (Table 1) was based a 1884 bp superlocus composed of the concatenated sequences of the loci for each strain in the following order: *ligB*-*secY*-*rpoB*-*lipL41*.

## Results

### Phylogenetic analysis of the *ligB*, *secY, rpoB* and *lipL41* loci

**Figure 1:**
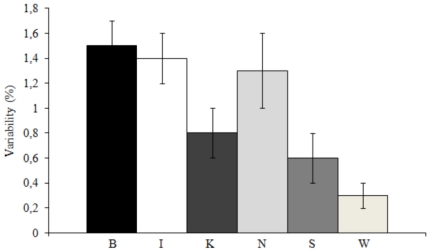
Alignment analysis of the concatenated superlocus. Comparison of the variability of the DNA sequences from the *ligB*-*secY*-*rpoB*-*lipL41* superlocus among the *Leptospira* species included in this study, where B – *L. borgpetersenii*, I – *L. interrogans*, K – *L. kirschneri*, N – *L. noguchii*, S – *L. santarosai* and W – *L. weilii*. Results are shown as means ± SD. The nucleotide positions used during the alignment analysis were: nt 2236–2449 (*ligB*), 771–1015 (*secY*), 1922–2462 (*rpoB*) and 73–956 (*lipL41*) and refer to the *L. interrogans* serovar Copenhageni L1-130 strain.

**Figure 2:**
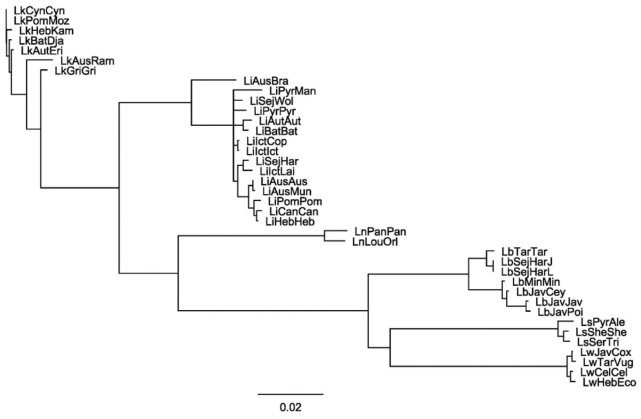
Phylogenetic analysis of 38 *Leptospira* serovars. The tree was constructed based on the *ligB*-*secY*-*rpoB*-*lipL41* superlocus sequences. The loci were analyzed using the Neighbor-Joining method as implemented in Geneious Pro 4.7.5 [25]. The samples are represented by the serovar followed by the strain designations. Confidence in the topology of this tree was gauged by bootstrap resampling (1,000 times).

The main criteria to select genes for the presented MLST scheme were their ability to separate one or more species in different clusters and to discriminate the strains and clinical isolates within them. To identify candidate loci with these properties we searched those previously characterized by the authors and mined public databases to obtain additional representative sequences. The genes used to constitute this study (*ligB*, *secY*, *rpoB* and *lipL41*) showed an optimal discriminative power. Following alignment, the percentage of identical pairwise amino acids residues (PI) for the *lipL41* locus was 92.0% and the percentage of identical sites (IS) among the DNA sequences was 77.9%, the *rpoB* locus was 91.8 and 75.2%, the *secY* locus was 87.8 and 71.4%, and the *ligB* locus was 82.0 and 48.6%, respectively. When all four loci for each strain were concatenated and when the resulting superloci were aligned the overall PI was 90.3% and the IS was 72.9%. Of note, intraspecies identity was considerably higher, 97.7–98.5±0.2% (Figure 1). The phylogenetic tree formed two major clusters representing *L. kirschneri* and the other species: *L. interrogans, L. noguchii, L. borgpetersenii*, *L. santarosai* and *L. weilii* (Figure 2).

The 15 *L. interrogans* strains, two *L. noguchii* strains, four *L. weilii* strains and three *L. santarosai* strains, listed in Table 1, could all be discriminated using the superlocus and of the seven *L. kirschneri* strains, only two proved to be identical at the sequence level (strains 5621 and 3522C). Among the seven *L. borgpetersenii* strains, the superlocus sequence was capable of discriminating all but two strains (JB197 and L550). Polymorphic sites, where one sampled sequence exhibits a unique base relative to the common nucleotide of the others were observed in 13 serovars and all species except *L. weilii*. The *L. interrogans* species included the largest number of polymorphic-containing serovars, six, followed by Pomona with two, then Autumnalis, Lai, Manilae, Muenchen and Pyrogenes with one. The *L. noguchii* serovars contained the largest number of unique polymorphic sites per serovar, Orleans and Panama had five each). Inclusion of the low polymorphic genes *rrs2* and *lipL32* as in the scheme of Ahmed and colleagues [18] was assessed but showed no advantage to the presented scheme (results not shown).

### Increased discrimination using the *ligB*, *secY*, *rpoB* and *lipL41* loci in an existing MLST scheme

**Figure 3:**
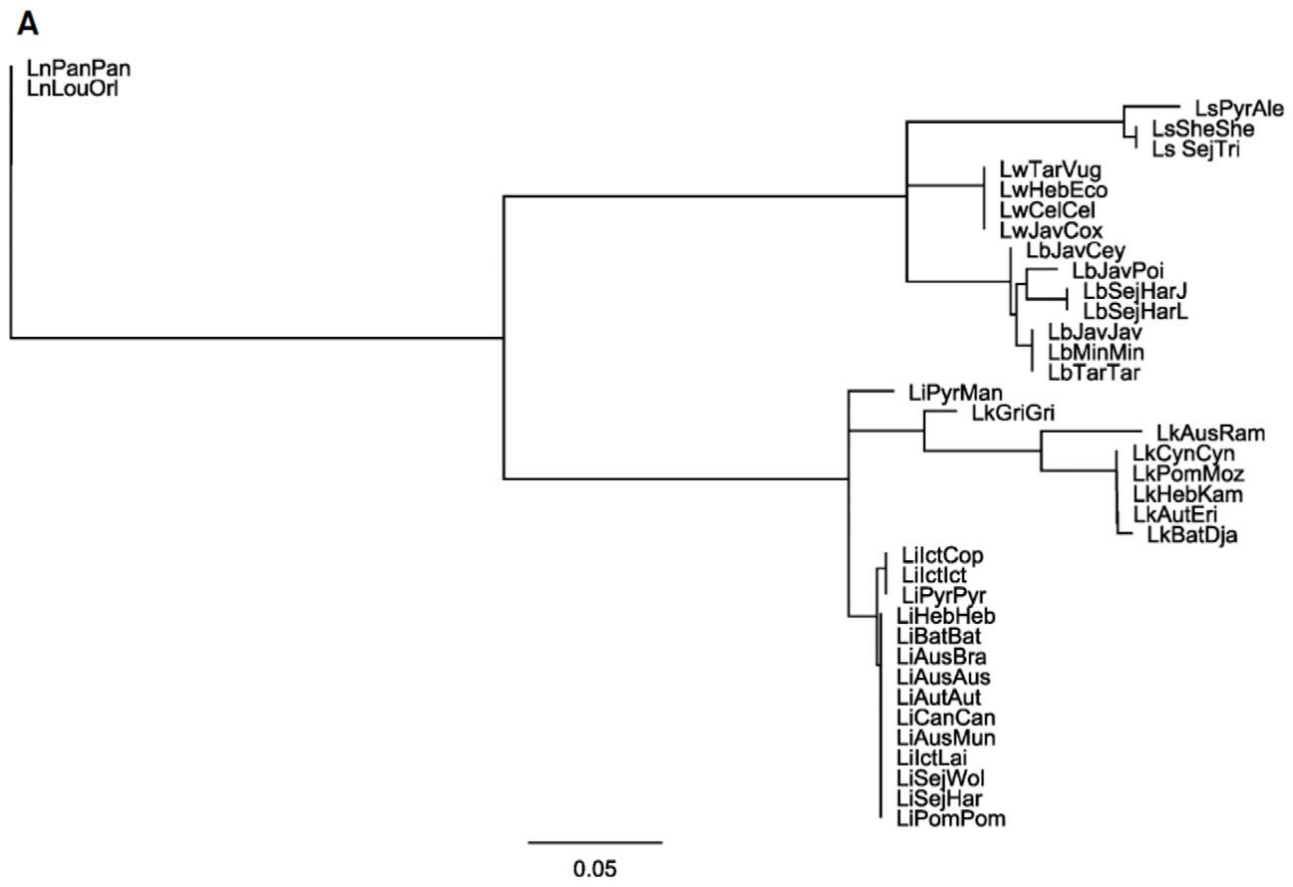
Analysis of the loci sequences from 38 *Leptospira* reference strains. The tree was constructed based on the individual loci sequences. The loci were analyzed using the Neighbor-Joining method as implemented in Geneious Pro 4.7.5 [25]. The samples are represented by the serovar followed by the strain designations. Confidence in the topology of this tree was gauged by bootstrap resampling (1,000 times). A: *ligB* loci; B: *secY* loci; C: *rpoB* loci; D: *lipL41* loci. Phylogenetic analysis was used to demonstrate the number of alleles that were distinguished for each loci A: *ligB* (15); B: *secY* (16); C: *rpoB* (19); and D: *lipL41* (27). Parts B–D can be found as supporting information in Figure S1.

**Figure 4:**
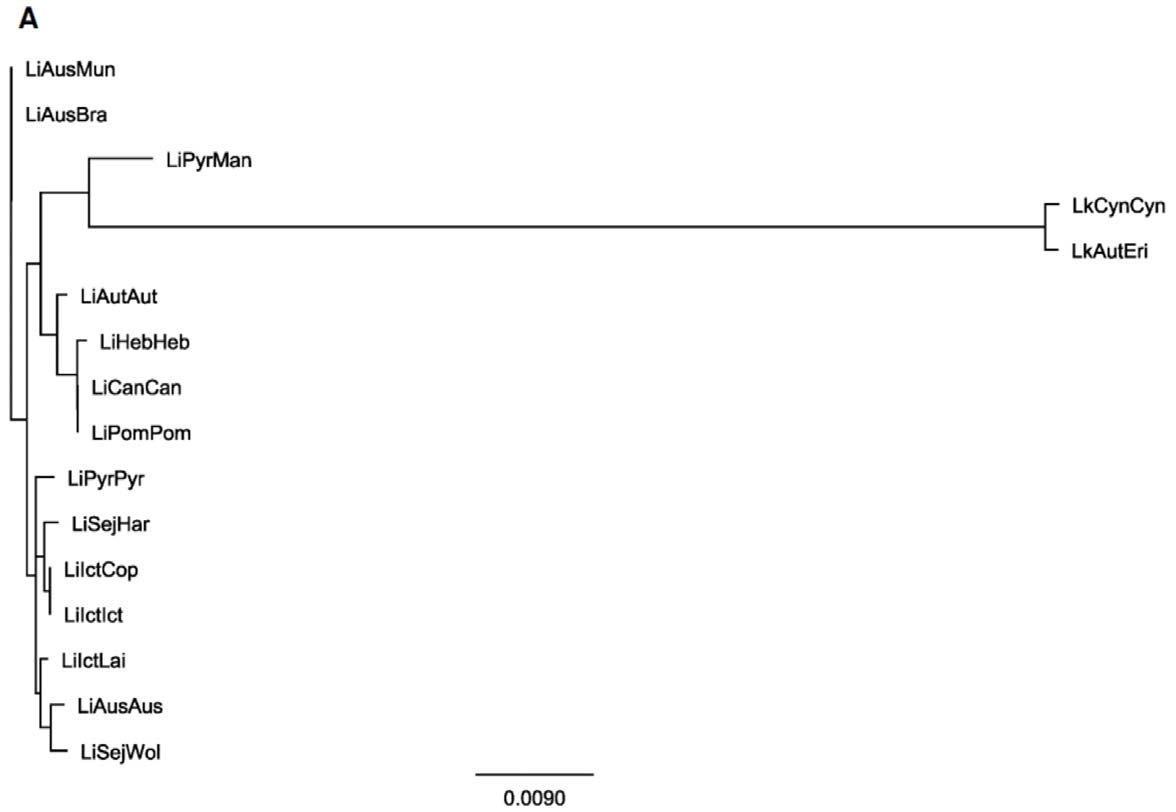
Phylogenetic analysis of the 16 ST from *L. interrogans* and *L. kirschneri*. The STs determined by Thaipadungpanit and colleagues [17] were concatenated with the loci analyzed during this study to test the effect of different loci combinations over serovars discrimination: (A) ST; (B) ST+*ligB*; (C) ST+*secY*; (D) ST+*rpoB*; (E) ST+*lipL41* and (F) ST+*rpoB*+*lipL41*. The tree was constructed by the Geneious Pro 4.7.5 software [25] as previously described. Confidence in the topology of this tree was gauged by bootstrap resampling (1,000 times). Parts B–F can be found as supporting information in Figure S2.

Based on analysis of the loci sequences from 38 *Leptospira* reference strains, *ligB* contained 15 alleles, *secY* 16 alleles, *rpoB* 19 alleles and *lipL41* 28 alleles, Figure 3 and Figure S1 B–D, respectively. Using the online *Leptospira* MLST scheme (http://leptospira.mlst.net, [17]) we identified 16 sequence types (ST), out of 109 ST, where the corresponding *ligB*, *secY*, *rpoB* and *lipL41* sequences for each strain were readily available. Using the concatenated sequences that corresponded to the *glmU*, *pntA*, *sucA*, *fadD*, *tpiA*, *pfkB* and *mreA* loci a phylogenetic tree was constructed that identified 13 unique ST among the 16 different strains included in the analysis (Figure 4). The *ligB*, *secY*, *rpoB* and *lipL41* loci were added to the MLST superloci from each strain to determine their impact on the level of discrimination. While inclusion of the *ligB* locus did not improve upon the original scheme (Figure S2 B), the *secY* and *lipL41* loci resolved 14 ST (Figures S2 C & D, respectively) and the *rpoB* locus discriminated between 15 ST (Figure S2 E). All possible combinations of the candidate loci were used to create additional superloci to determine the assembly with the greatest discriminatory power (data not shown). Complete resolution of the 16 ST was achieved by inclusion of both *lipL41* and *rpoB* loci in the original concatenated sequence of each strain, Figure S2 F.

## Discussion

The MLST scheme proposed by Thaipadungpanit and colleagues is based on loci from seven housekeeping genes and identified 109 unique ST among 263 isolates from either *L. interrogans* or *L. kirschneri* [17]. This represented a major advance in the molecular epidemiology of *Leptospira* isolates. Unfortunately, as noted by the authors themselves, a major limitation of this scheme is that it does not allow for the inclusion of the other common pathogens associated with human leptospirosis [20]. Previously, it was shown that the antigen encoding genes *ligB*, *secY*, *lipL41* and the *rpoB* gene are potentially useful for the molecular discrimination of *Leptospira* strains and that they can be readily amplified from all known pathogenic leptospires [23]–[25], [29], [30]. Furthermore, the authors of the original bacterial MLST scheme recommend the inclusion of loci from antigen coding genes to improve discrimination, especially during outbreak investigations [31].

To determine the potential benefits of using *ligB, secY, rpoB* and *lipL41* loci in an MLST typing scheme focused on strain differentiation we identified the corresponding sequences in a reference collection containing 38 *Leptospira* strains (Table 1). The overall level of pairwise identity ranged from 82–92% among the individual loci while the intraspecies identity was even higher (Figure 1). Furthermore, when the sequences were concatenated to create a superlocus for each strain and analysed, the overall pairwise identity was >90%. Following the modelling of phylogenetic trees and in agreement with previous studies, two distinct evolutionary branches were observed, the first contained *L. kirschneri*, *L. interrogans* and *L. noguchii* strains and the second the *L. borgpetersenii*, *L. santarosai* and *L. weilii* strains (Figure 4 and Figure S2) [23], [29]. There was some evidence that serovars from the same serogroup clustered together, serogroups: Icterohaemorrhagiae: *L. interrogans* Icterohaemorrhagiae RGA and Copenhageni Fiocruz L1-130; Australis: *L. interrogans* Australis Ballico and Muenchen Muenchen C90; and Javanica: *L. borgpetersenii* Javanica Veldrat Batavia 46, Poi Poi and Ceylonica Piyasena (Figure 2). This may indicate homoplasy (similarity due to convergent evolution) of the genes from these serovars. This analysis showed a separation of serovar Bratislava from the other *L. interrogans* serovars (Figure 2). Alignment analyses demonstrated this is probably due to the high similarity of the *L. interrogans* Jez Bratislava strain *rpoB* gene sequence with those from the *L. borgpetersenii* strains (data not shown). Despite this, serovar Bratislava was correctly located within the *L. interrogans* clade. We did not determine however, whether this was due to sequence mosaicism or horizontal gene transfer. The phylogenetic organisation of the *Leptospira* genus based on the superlocus supports the theory [29] that *L. interrogans* is more recently evolved from *L. kirschneri*, more recent evolutionary subdivisions resulted in the separation of *L. borgpetersenii* followed by *L. santarosai* and *L. weilii* clades.

Analysis of the discriminatory power of the *ligB-secY-rpoB-lipL41* superlocus found that the *L. interrogans*, *L. noguchii*, *L. santarosai* and *L. weilii* strains could be separated into individual ST. However, among the *L. kirschneri* and *L. borgpetersenii* strains, two could not be resolved by the superlocus. Serological analysis of the two *L. borgpetersenii* strains indicated that both belonged to serogroup Sejroe serovar Hardjo-bovis and their genomes were found to be highly conserved. Yet they are distinct clonal subtypes, both strains established chronic infections in cattle yet differed in their ability to cause lethal infections in hamsters [32]. Despite this no specific polymorphisms were observed in either of the *L. borgpetersenii* L550 or JB197 strains. These polymorphic regions are normally useful for surveillance purposes, to monitor outbreaks or for epidemiological studies. Thirteen serovars, out of 38 in this study, exhibited unique polymorphic sites. These findings highlight the efficiency of the proposed *ligB-secY-rpoB-lipL41* superlocus to discriminate *Leptospira* strains. The study previously performed by Ahmed and colleagues [18] was the pioneer in the use of a concatenated superlocus to discriminate among the *Leptospira* species. However, this work was intended as a step towards the study of pathogen evolution rather than strain discrimination. Inclusion of low polymorphic genes such as *rrs2* and *lipL32* used by Ahmed et al., [18] did not contribute to enhance the discriminative power of the MLST scheme presented here. In the present work, we included some of those sequences in combination with recently sequenced polymorphic genes to increase the resolution, and observed the occurrence of discrimination to the subspecies level. Although a reduced number of strains and isolates were included in our study we believe the proposed superlocus presents a solid basis for discriminating within large panels of *Leptospira* strains and isolates.

An analysis of the *ligB*, *secY*, *rpoB* and *lipL41* loci found that the *ligB* locus was the most conserved with 15 alleles, followed by *secY* with 16 alleles, *rpoB* with 19 alleles and *lipL41* with 28 alleles out of a potential 38 (Figure 3 and Figure S1). Following analysis of the 263 isolates (109 ST) contained in the *Leptospira* MLST database (leptospira.mlst.net), 16 strains (corresponding to 13 ST) were identified as having the corresponding *ligB*, *secY*, *rpoB* and *lipL41* loci sequences available (Figure 4). To determine the utility of the loci proposed in this study the relevant sequence for each individual locus was concatenated to the *glmU*, *pntA*, *sucA*, *fadD*, *tpiA*, *pfkB* and *mreA* superlocus of each strain (Figures S2 B–E). All possible variables were evaluated in order to identify the most useful additional loci. The combination of the original superlocus together with the *rpoB* and the *lipL41* loci was found to be the simplest superlocus that could discriminate between all 16 of the strains (Figure S2 F). This is in agreement with the ability of the superlocus determined by Ahmed and colleagues [18], which includes the *lipL41* locus, to discriminate the *Leptospira* spp. in study.

The phylogenetic analysis of our sequences showed a great diversity of ST and no clustering, due to the use of epidemiologically unrelated strains. Thus, when the two new loci sequences were concatenated to the original ST sequences we observed the complete discrimination of the strains, although our adapted scheme remains limited to *L. interrogans* and *L. kirschneri*. We recommend that the *rpoB* and *lipL41* loci be evaluated in existing or future MLST schemes to enhance their typing power during outbreak investigations.

## Supporting Information

Figure S1 Continued from figure 3.

Figure S2 Continued from figure 4.
