# Supplementary figures and images for: Bioinformatics Describes Novel Loci for High Resolution Discrimination of Leptospira Isolates
Source: PLoS One. 2010 Oct 15;5(10):e15335. doi: 10.1371/journal.pone.0015335 (PMC2955542; doi:10.1371/journal.pone.0015335)

FIGURE 3

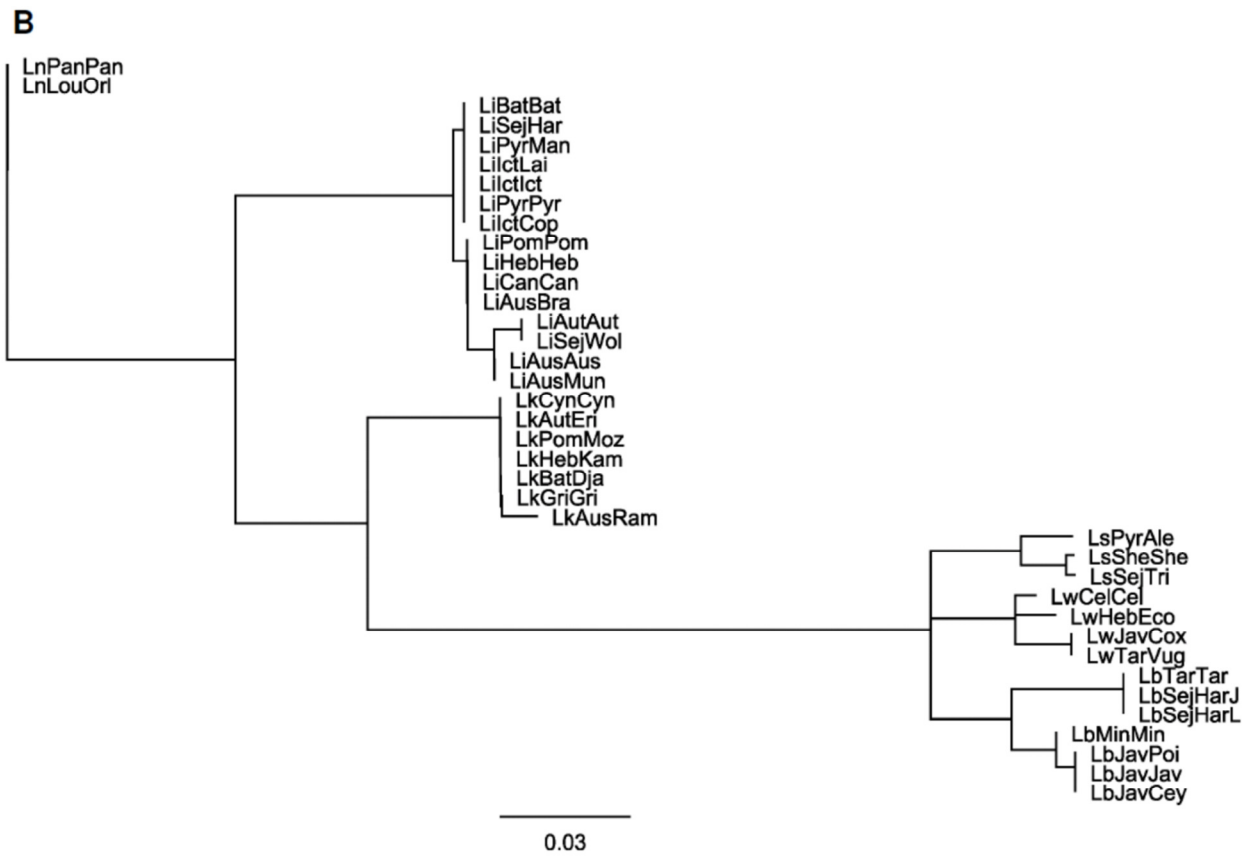

FIGURE 3

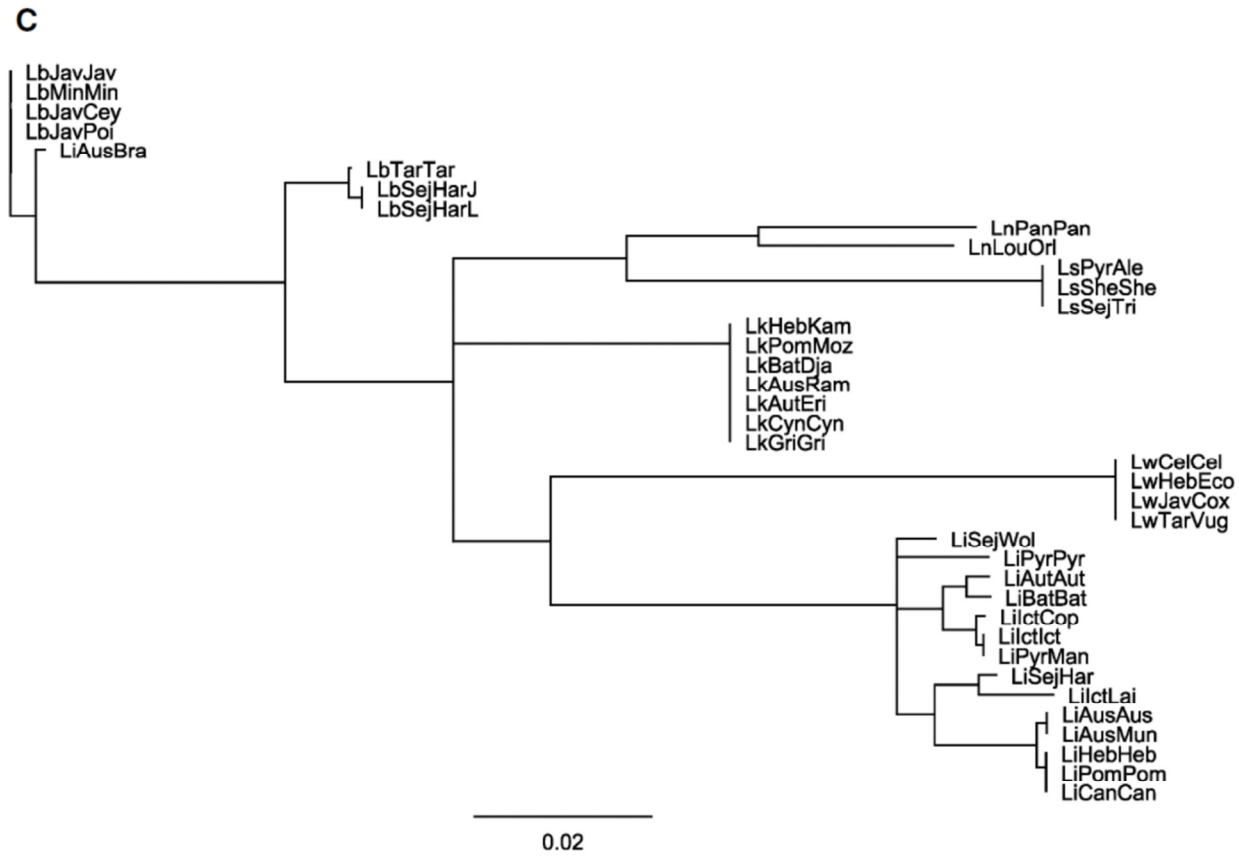

FIGURE 3

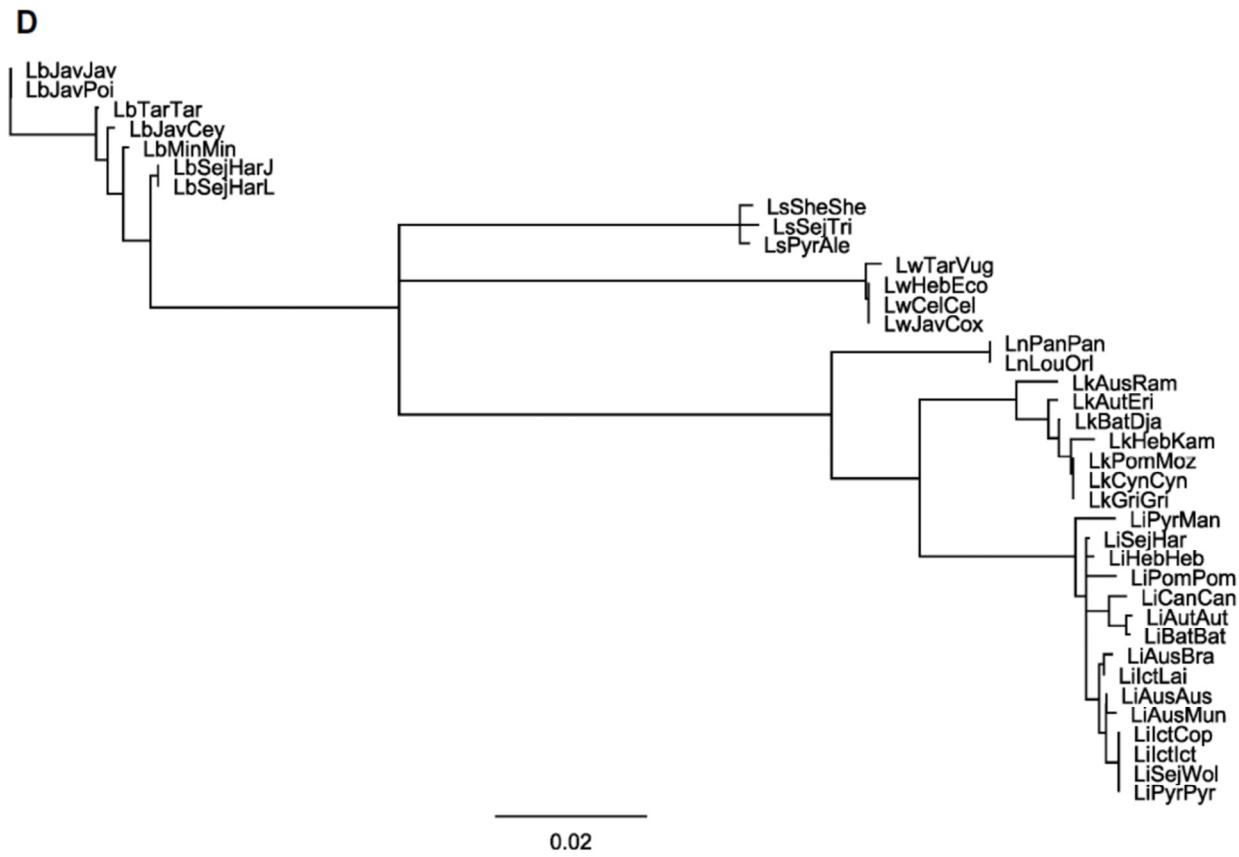

Supplement: Figure S1 — Continued from figure 3. [file pone.0015335.s001.pdf]

**FIGURE 4**

**B**

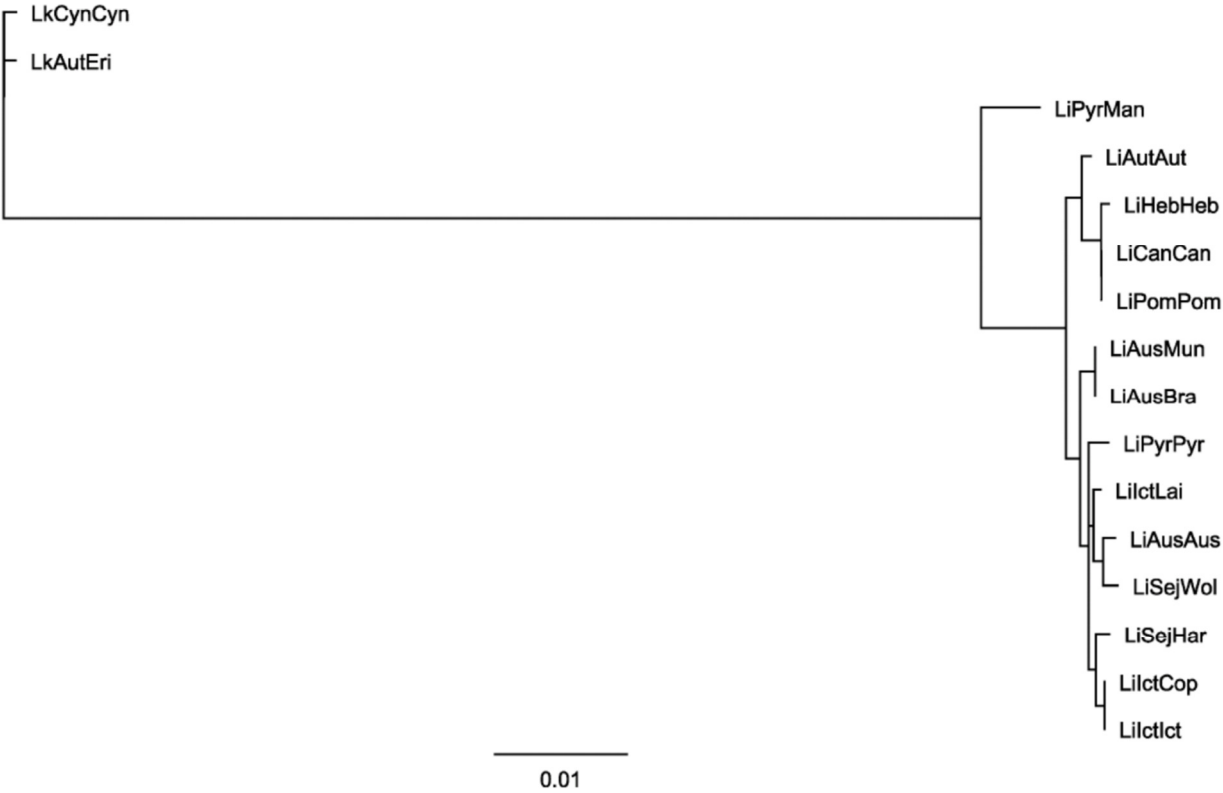

FIGURE 4

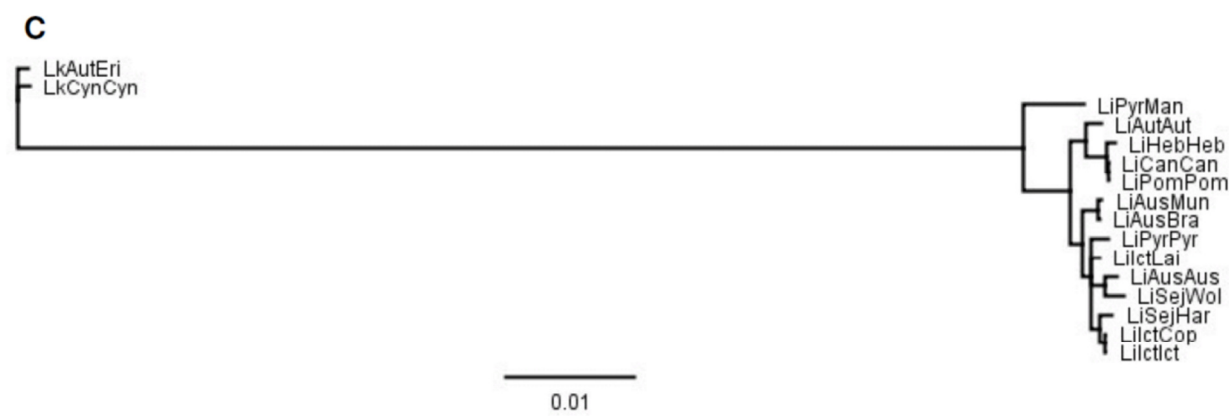

**FIGURE 4**

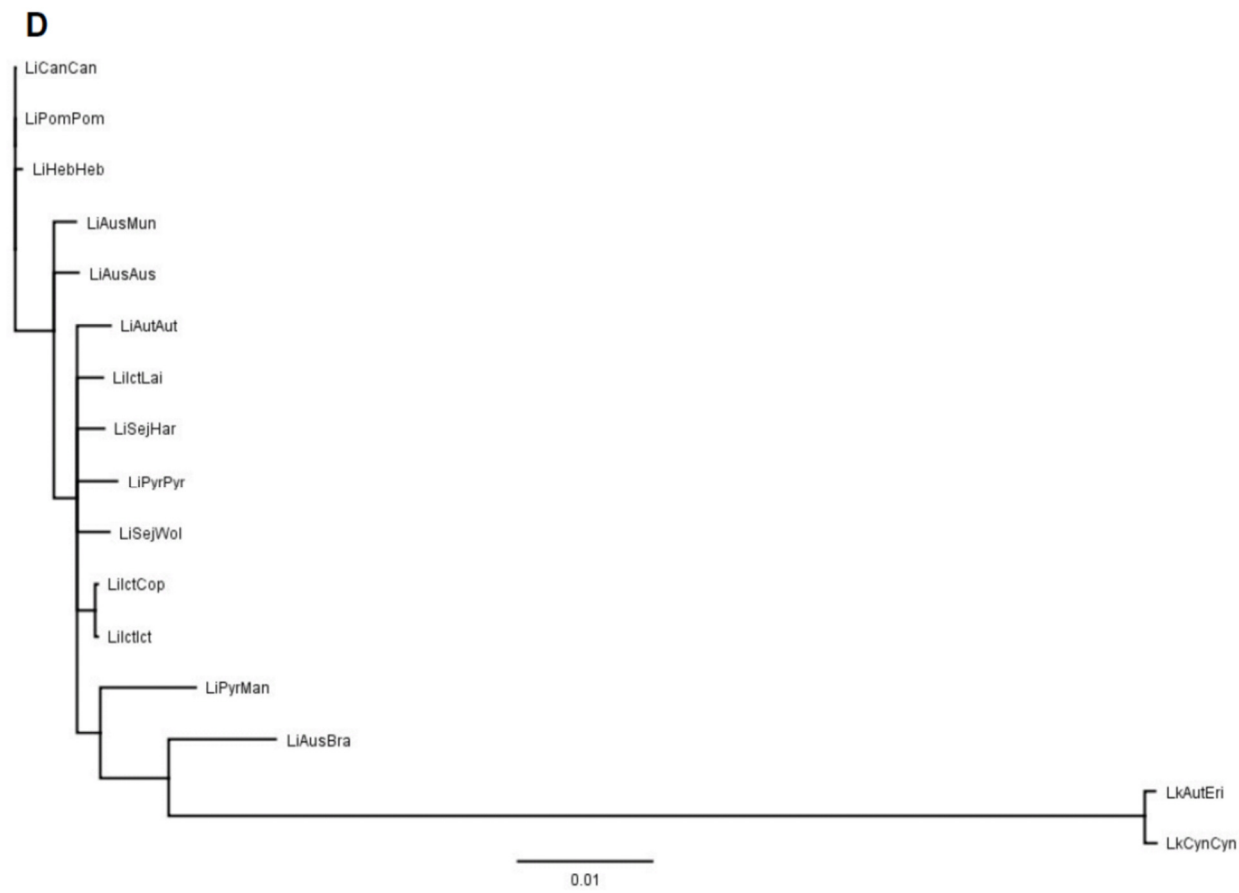

FIGURE 4

E

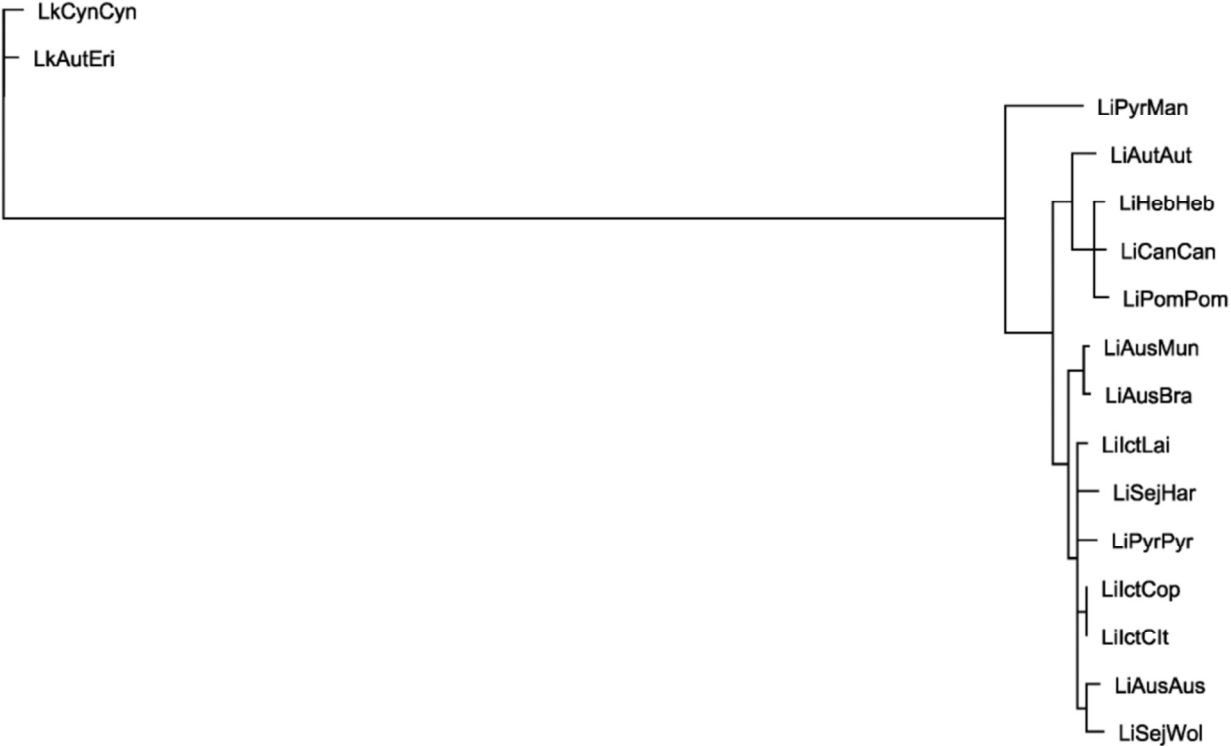

0.0090

FIGURE 4

F

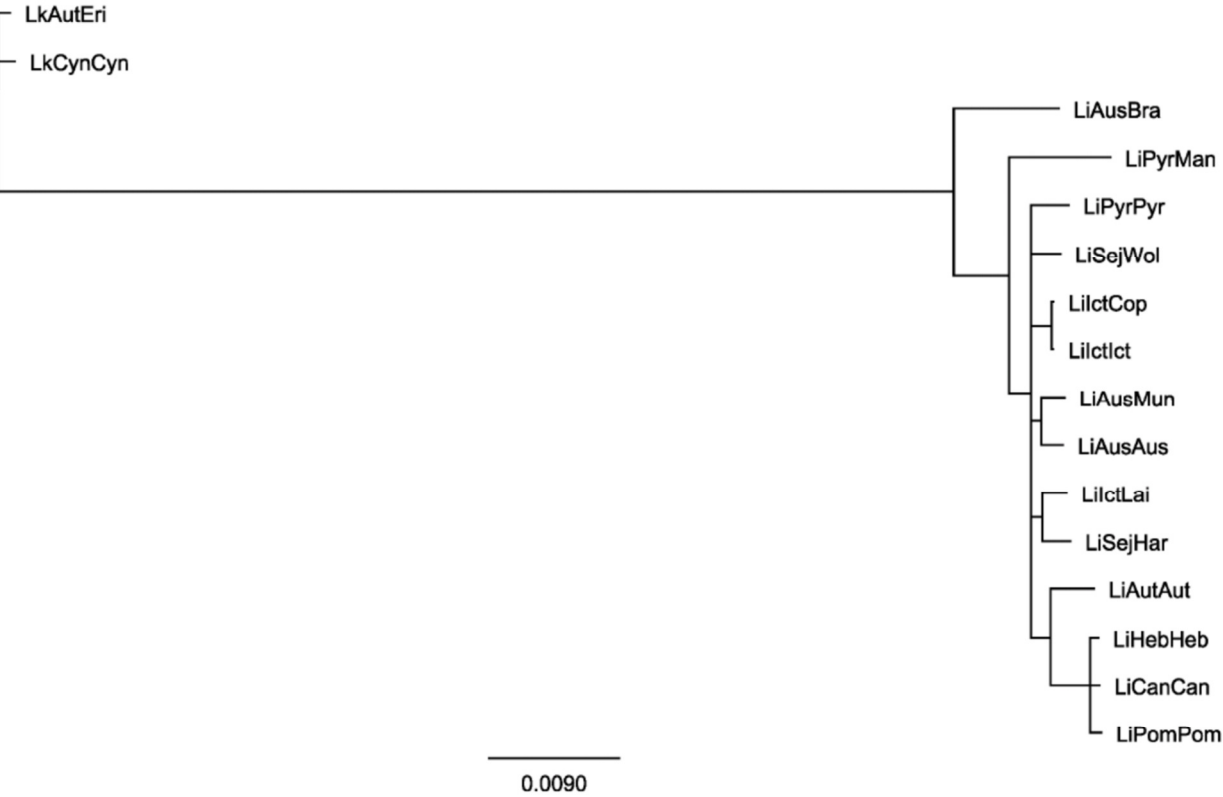

Supplement: Figure S2 — Continued from figure 4. [file pone.0015335.s002.pdf]
